# Outcomes following resuscitative thoracotomy for abdominal exsanguination, a systematic review

**DOI:** 10.1186/s13049-020-0705-4

**Published:** 2020-02-06

**Authors:** Michael Hughes, Zane Perkins

**Affiliations:** 1 Scarborough Hospital, York Teaching Hospital NHS Trust, Woodlands drive, Scarborough, YO12 6QL UK; 2 Queen Mary University, London, E1 4NS UK

## Abstract

**Background:**

Resuscitative thoracotomy is a damage control procedure with an established role in the immediate treatment of patients in extremis or cardiac arrest secondary to cardiac tamponade however Its role in resuscitation of patients with abdominal exsanguination is uncertain.

**Objective:**

The primary objective of this systematic review was to estimate mortality based on survival to discharge in patients with exsanguinating haemorrhage from abdominal trauma in cardiac arrest or a peri arrest clinical condition following a resuscitative thoracotomy.

**Methods:**

A systematic literature search was performed to identify original research that reported outcomes in resuscitative thoracotomy either in the emergency department or pre-hospital environment in patients suffering or suspected of suffering from intra-abdominal injuries. The primary outcome was to assess survival to discharge. The secondary outcomes assessed were neurological function post procedure and the role of timing of intervention on survival.

**Results:**

Seventeen retrospective case series were reviewed by a single author which described 584 patients with isolated abdominal trauma and an additional 1745 suffering from polytrauma including abdominal injuries. Isolated abdominal trauma survival to discharge ranged from 0 to 18% with polytrauma survival of 0–9.7% with the majority below 1%. Survival following a thoracotomy for abdominal trauma varied between studies and with no comparison non-intervention group no definitive conclusions could be drawn.

Timing of thoracotomy was important with improved mortality in patients not in cardiac arrest or having the procedure performed just after a loss of signs of life. Normal neurological function at discharge ranged from 100 to 28.5% with the presence of a head injury having a negative impact on both survival and long-term morbidity.

**Conclusions:**

Pre-theatre thoracotomy may have a role in peri-arrest or arrested patient with abdominal trauma. The best outcomes are achieved with patients not in cardiac arrest or who have recently arrested and with no head injury present. The earlier the intervention can be performed, the better the outcome for patients, with survival figures of up to 18% following a resuscitative thoracotomy. More high-quality evidence is required to demonstrate a definitive mortality benefit for patients.

## Background

The management of the peri-arrest or arrested patient following major trauma is controversial with poor outcomes in both civilian and military practice [1, 2]. Current guidelines advocate initiating cardiopulmonary resuscitation, intubation and bilateral thoracostomies followed by rapid transfer to theatre and if available, a range of advanced procedures including a thoracotomy for specific injuries [3].

A thoracotomy is referred to by a variety of names in peri-arrest or arrested patients depending on the physical location it is performed and the physiological status of the patient (e.g. resuscitative thoracotomy, emergency department thoracotomy etc) [4, 5]. It is the subject of intense debate and its role within trauma management, especially in blunt trauma, is not certain. However, the procedure is included in the European resuscitation council guidelines for traumatic cardiac arrest for both penetrating and blunt trauma with survival rates of 6–7% reported by several studies for those suffering from penetrating chest injuries, demonstrating the best outcomes [6–8].Cross-clamping of the thoracic aorta in order to arrest bleeding and improve blood flow proximal to the clamp is a recognised part of the procedure [9]. This is a manoeuvre used both in and out of the operating theatre and is specifically applied to those with intrabdominal trauma in some circumstances, gaining proximal control of arterial bleeding via the chest cavity [9]. The role of a thoracotomy in abdominal trauma assumes that arrest is secondary to hypovolemia due to ongoing haemorrhage and thus by temporarily controlling this before definitive management can be instigated, patient outcomes can be improved. This theory has been reinforced by reports that traumatic cardiac arrest is a “low flow state” rather than a cessation of cardiac activity suggesting that rapid intervention to control haemorrhage may be of benefit [10].

Although patient’s with abdominal trauma presenting to UK hospitals have benefited from improved mortality following the introduction of major trauma networks and centralisation of specialist services, survival in this subset of peri arrest patients continue to be poor [11]. It has been demonstrated that patient’s requiring a laparotomy that present to hospital hypotensive have poor outcomes. Mortality as high as 46% has been reported, with prolonged time to theatre with ongoing hypotension worsening mortality further. However, the role of a thoracotomy in abdominal trauma has not been fully explored [12–14].

Timing of resuscitative thoracotomy is important as there is a demonstrated correlation between early intervention and improved mortality [15]. Protocols have been developed for those in traumatic cardiac arrest with a 10 min “cut off” from loss of signs of life, after which a thoracotomy should not be performed, being used by some institutions such as the London Helicopter Emergency Medical Service [16]. Guidelines differentiate between blunt and penetrating trauma advocating a 10–15-min window for intervention depending on the mechanism of injury [3]. A resuscitative thoracotomy can be performed in both the pre-hospital and emergency department setting giving the potential for the intervention to be performed earlier outside of an operating theatre.

Although survival from a procedure is the ultimate end point, the quality of life for patients afterwards is also essential to consider. Neurological outcomes following resuscitative thoracotomy are variable. In patient undergoing the procedure in the pre-hospital environment following penetrating trauma after a short period of time in cardiac arrest outcomes were described as good however, in some cases patients never recover normal neurological function [17–19].

Comparing the outcomes of a thoracotomy to no thoracotomy in abdominal trauma is difficult as little data is available. The Eastern Association for the Surgery of Trauma have made estimates of survival based on expert opinion [20]. Survival varies depending on the presence of signs of life and mechanism of injury (penetrating Vs blunt). In patients presenting to the emergency department with a pulse and evidence or suspicion of penetrating extra thoracic trauma the expert working group suggest that the chance of survival following an emergency thoracotomy was 15.6% compared to 1.7% without. Patient’s with the same pattern of injury but arriving without a pulse had an estimate average survival of 2.9% following a thoracotomy and 0.1% without.

In blunt trauma the expert group estimated that patient survival was much lower when suffering from an extra thoracic pattern of injury when compared to penetrating trauma but still felt emergency thoracotomy made a difference to survival. In those arriving with a pulse, survival following a thoracotomy was 4.6% compared to 0.5% without. Patient arriving without a pulse following blunt trauma had an estimated survival of 0.7% with a thoracotomy compared to 0.001% without.

The aim of this systematic review was to estimate the overall change in mortality for adults with clinically diagnosed exsanguinating abdominal haemorrhage treated with resuscitative thoracotomy before entering theatre, based on available evidence and then compare this with the expert group estimates of survival.

### Hypothesis

Resuscitative thoracotomy does not improve mortality, quantified as survival to discharge, in blunt or penetrating abdominal trauma in contrast to expert opinion estimating outcomes following current conventional management.

### Aims

- Identify current use of pre-hospital and emergency department resuscitative thoracotomy in intra-abdominal trauma (either isolated or part of multiple injuries)
- Evaluate overall outcomes in intervention group and focus specifically on survival to discharge with secondary assessment of timing of intervention and neurological outcome in survivors.
- Contrast outcomes of patient’s undergoing resuscitative thoracotomy with expert opinion of survival with patient’s suffering similar injuries but not having the intervention.

### PICOS

**P** Patient with suspected haemorrhagic shock secondary to intra-abdominal trauma (either isolated or as a combination of injuries) in a peri-arrest or arrest clinical condition requiring immediate resuscitation and intervention.

**I** Resuscitative thoracotomy (before entering operating theatre).

**C** Survival based on estimated opinion of standard care (tranexamic acid, rapid transport to theatre including an in-theatre thoracotomy).

Mortality (survival to discharge), role of timing of intervention, neurological outcome.

**S** A systematic review of current literature evaluating resuscitative thoracotomy (defined as a thoracotomy performed either in the pre-hospital environment of emergency department) for patient with intra-abdominal injuries.

## Methods

This systematic review was conducted in line with the Preferred Reporting Items for systematic reviews and Meta-Analysis statement [21].

### Search strategy

A comprehensive literature review was conducted by a single author (M.H) which was completed on 24/1/2018. A search of the electronic databases Medline and Embase was conducted using medical subject heading (MESH) terms shown below with the Cochrane database and PROSPERO also being used to identify any completed or ongoing systematic reviews on the subject matter [22]. Finally, Google scholar was searched using broad, non MESH search terms.

- Pre-hospital
- Emergency department
- Ambulance
- Paramedic
- EMS
- Out of hospital
- Abdominal trauma
- Blunt
- Penetrating
- Wound
- Injur*
- Thoracotomy
- Resuscitative thoracotomy
- Emergency thoracotomy
- Trauma

Papers were screened and reviewed based on the eligibility criteria outlined below by a single reviewer. Title of paper, abstract and if required full paper review was used to assess for inclusion. All references in papers were followed up and included where relevant and authors were contacted if any information was unclear.

### Eligibility criteria

All types of studies were included apart from expert opinion, single case reports and review articles (systematic reviews, meta analysis, systematic review). Studies before 1987 were excluded as it was felt that data from more than 30 years ago would not be relevant to current medical practice. Foreign language papers were also excluded as no interpreter was available to ensure any translations were accurate and precise. There was no exclusion on age and therefore any paediatric data was included.

Table 1 gives a summary of inclusion and exclusion criteria. Additional file 1: Appendix 2 contains this summary as well as the perceived weakness these criteria generated.

**Table 1:** A table to show the exclusion and inclusion criteria for the study protocol

	Inclusion	Exclusion
Study design	All original research study designs were be included. Expert opinion and case studies were excluded meaning all except level 5 and 4 evidence will be included as defined by the oxford centre for evidence-based medicine [23]. As only original research was reviewed systematic reviews and literature review was also excluded	Expert opinion Case studies Literature review Systematic review Editorials
Study specific details	After 1987	Non-English language Study pre-1987 Duplicates
Quality of evidence	Will be evaluated by all included	
Resuscitate thoracotomy pre-theatre	Procedure must be performed in the pre-theatre environment (ED or pre hospital)	In theatre
Abdominal trauma	Must have stated injuries of patients based on either clinical assessment or in hospital / pre-hospital imaging	Isolated chest/ pelvis
Outcomes	Must state outcomes of patients in terms of survival to destination or discharge. All studies were included if they included either the primary of secondary outcomes aims.	No patient outcomes included
Thoracotomy	Any thoracotomy (clamshell / left lateral)	Non-thoracotomy interventions e.g. REBOA

A single author (MH) evaluated each paper using the CASP checklists and the Cochrane handbook for systematic reviews to assess for validity and reliability [22, 24].If a paper did not answer the first 2 points of the CASP checklist satisfactorily it was marked as a poor-quality study but was still included (see Additional file 1: Appendix 3). The data was extracted by a single reviewer (MH) with all papers being reviewed on two separate occasions to ensure no data was overlooked or misinterpreted.

The following data was extracted for each study which is summarised in Table 1; Title, description of subjects, number of participants, study eligibility criteria, indication for thoracotomy, definition of traumatic cardiac arrest, study design, survival / mortality, neurological outcome, location of intervention (eg emergency department), authors conclusion, timing of intervention.

## Results

**Fig. 1:**
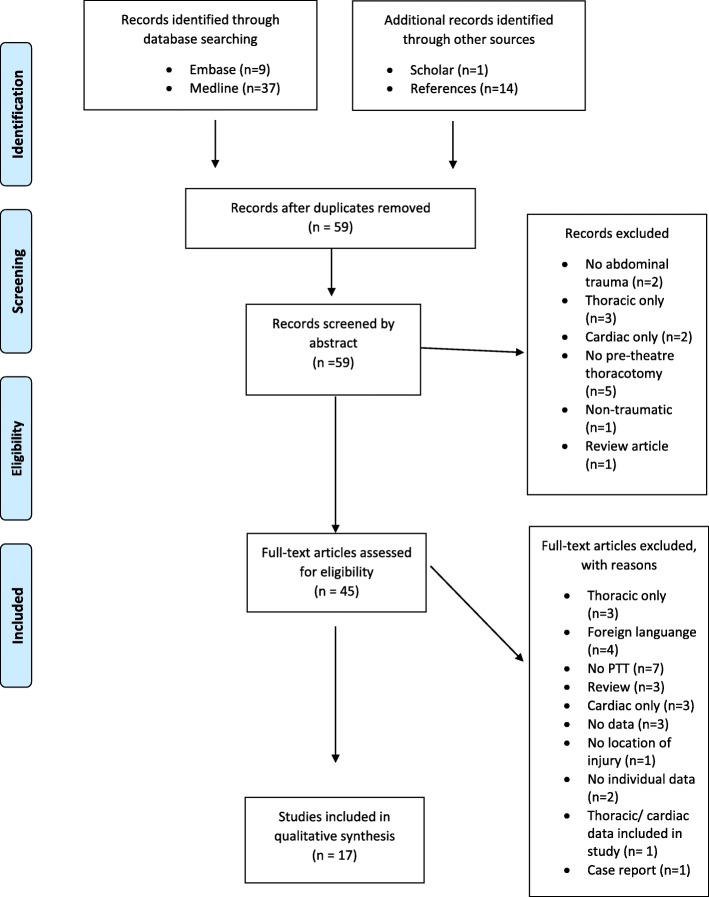
PRISMA outcomes from literature review

Figure 1 demonstrates the Preferred Reporting Items for systematic reviews and meta-analyses (PRISMA) flow diagram for this systematic review.

Seventeen retrospective case studies were included in the review. No meta-analysis was attempted due to the heterogenicity of data both methodological and clinical. Clinically some institutions had clear protocols for performing a thoracotomy but not all. Recording of the timing of intervention as well as the patient state at the start of the procedure (e.g. what constituted a loss of signs of life) and clear details of survival to discharge were also variable between studies.

### Indications and inclusion

There was wide variation in the indications for resuscitative thoracotomy with no pre-hospital thoracotomies being included in the review. The indications, exclusion and inclusion criteria for each study are summarised in Additional file 1: Appendix 3. Indications for thoracotomy included patient’s in cardiac arrest or who had recently arrested for all types of traumatic injury. There were also differences in timing of thoracotomy being performed ranging from immediately after the onset of cardiac arrest to over 35 min after. There were several terms used to suggested cardiac arrest in patients including loss of signs of life and the presence of fixed dilated pupils however even these terms were poorly defined with difference between studies as to what constituted a loss of signs of life [25].

#### Survival to discharge

The range of survival to discharge for patients undergoing a resuscitative thoracotomy before entering theatre in all studies was 0–16%. This increased to 18% of 22 patients if only an isolated iliac injury was present [26]. Table 2 summarises all the primary and secondary outcomes. Survival appeared to improve based on the mechanism of injury. The studies that reported survivors demonstrated broadly the same outcomes with isolated abdominal trauma having a better survival rate than polytrauma, penetrating having better outcomes than blunt, and the timing of intervention based on the presence of signs of life, being important.

**Table 2:** A Table to summarise the number of patient’s undergoing a resuscitative thoracotomy, the number and percentage (%) of patients that survived, any comment on neurological outcome and the role of timing of the intervention for each included study

Title	Number of patients undergoing pre-theatre thoracotomy	Number of patients surviving to discharge	% of patients survival to discharge	Neurological outcome (if commented on)	Timing of intervention (if commented on)
Velmahos 1995 [27]	Isolated abdomen 118 Polytrauma 501	Isolated abdomen 8 Polytrauma 1	7% Less than 1%	No data	Best outcomes with witnessed loss of signs of life
Asensio 2003 [26]	22	4	18%	No data	No data
Asensio 2005 [28]	3	0	0%	No data	No data
Blocksom 2004 [29]	27	3	11%	No data	No data
Kalina 2009 [30]	Isolated abdomen 7 Polytrauma 13	? – unclear	No data	No data	Presence of signs of life in field best predictor of survival
Moore 2016 [31]	Isolated abdomen 116 Polytrauma 1003	Isolated abdomen 7 Polytrauma 371	6% 37%	68% no permanent neurological deficit with 12% mild neurological deficit and remaining 20% in a persistent vegetative state	No Pre-hospital CPR being performed associated with better chance of survival
Asensio 2007 [32]	4	0	0%	No data	No data
Ross 1988 [33]	7	0	0%	No data	No data
Nicholas 2003 [34]	7	0	0%	No data	No data
Mazzorana 1994 [35]	252	4	1.6%	No neurological deficit	Improved survival if signs of life at time of thoracotomy
Tyburski 2001 [25]	31	2	6%	No data	No data
Moore 2015 [36]	72	4	5.5%	28.5% chance of no neurological deficit.	No data
Seamon 2008 [37]	50	8	16%	No neurological deficit	No data
Asensio 2001 [38].	180	50	28%	No data	Spontaneous breathing at time of procedure associated with better chance of survival
Asensio 2000 [39].	43	1	2%	No data	No data
Branney 1998 [40].	Penetrating abdominal 73 Blunt abdominal 51	8 1	10% 2%	No neurological deficit	Better outcomes if signs of life present in pre-hospital environment
Lustenberger 2012 [41].	31	4	13%	No data	No data

The widest variation in survival to discharge rates was found in patients with blunt trauma, although It was difficult in several studies to differentiate whether this was isolated abdominal trauma or combined with pelvic trauma and injuries to other locations.

#### Timings of intervention

None of the studies included looked specifically at timing of intervention from onset of cardiac arrest or injury and the effect on mortality. Several commented on the improved mortality of those who’s arrest was witnessed or occurred within the emergency department, but none were tested and found to be statistically significant. On-going CPR was generally a negative predictor of survival in both penetrating and blunt trauma [31]. Several studies demonstrated that the presence of a pulse or even pulseless electrical activity was a positive predictor of survival [35, 37, 38].

#### Neurological outcome

Neurological outcome in patients undergoing a pre-theatre thoracotomy was explored in three studies with a wide variety of outcomes. The first study of 252 patients had 4 survivors none of which had any neurological deficit after undergoing pre-theatre thoracotomy for penetrating abdominal and pelvic traumatic injuries [35]. The most negative figure was in a study that looked at patients discharge destination and inferred their neurological status based on this. One of four survivors undergoing a pre-theatre thoracotomy had no neurological deficit at time of discharge from hospital [36]. A study of 106 patients who survived a thoracotomy demonstrated that 68% had no permanent neurological deficit,12% had a mild neurological deficit and the remaining 20% were in a persistent vegetative state although this did not separate into abdominal injuries only.

## Discussion

### Survival to discharge

Unfortunately, there was a small number of studies regarding the use of resuscitative thoracotomy for abdominal trauma and the overall quality of the evidence was poor. The initial PICO question focused on contrasting the current management of the peri-arrest or recently arrest trauma patient with abdominal trauma with that of the information gathered from the systematic review. The only information that could be found on outcomes following best current management of abdominal trauma was expert opinion which it is well documented is open to a bias. A systematic review and meta-analysis on the use of emergency department thoracotomy in blunt trauma concluded 1.5% of patients survived this intervention following a loss of signs of life with the majority having poor neurological outcome at discharge but did not look at specific location of injury [42]. This study still suggested that there may be a role for resuscitative thoracotomy with the chance of survival without it being so poor.

Assuming the expert opinion is accurate then the best probability of survival to discharge for patients with abdominal injuries was in the group who were suffering from unrelenting shock but with a pulse present following penetrating trauma. Based on current management, without a resuscitative thoracotomy before entering an operating theatre survival was 1.7%. All other patterns of injury and worsening states of shock including cardiac arrest had even lower survival to discharge figures without a resuscitative thoracotomy. This is in a population predominantly of young, fit individuals with trauma being the commonest cause of death in the UK within the ages of 1–40 [43]. This can be compared to the survival to discharge from medical cardiac arrest in the UK which is 8.6% [44]. The last major intervention to improve mortality in trauma care was the widespread use of tranexamic acid in trauma patients which although an undoubted break through still leaves an unacceptably high mortality particularly in the context of managing young, healthy individuals [45].

The use of pre-laparotomy thoracotomy in theatre was strongly advocated by several studies as a method of improving overall survival and was proven to be statistically significant [25, 38]. This suggests that the intervention its self with cross clamping of the aorta is effective but perhaps it is the timing based on the patients physiology that is important.

A literature review including 640 patient’s with isolated abdominal injuries and 590 patient’s suffering from polytrauma undergoing a resuscitative thoracotomy before theatre suggested similar findings to those in the systematic review [46]. It reported a survival of 4.5% in isolated abdominal injury and 0.7% in polytrauma.

The presence and loss of signs of life was explored in most studies suggesting the importance of the patients clinical condition at the time of intervention. Although there was wide variety of practice in the indications and use of resuscitative thoracotomy, the majority were performed once a patient had lost cardiac output. This clearly has a bearing on the interpretation of the results as the majority of patients suffering a traumatic cardiac arrest are highly unlikely to survive making even small improvement in mortality significant. Other large studies looking at resuscitative thoracotomy for blunt trauma have suggested the lack of electrical activity is a negative predictor of mortality and no intervention should be performed if cardiac electrical activity is absent [47].

There is no unifying definition of a resuscitative thoracotomy with varying terms for thoracotomies performed within the emergency department making the initial literature search problematic leaving the potential for some studies to have been omitted, thus affected the overall reliability and validity of the study. Another consideration not assessed was clinicians discretion to perform intervention based on other factors such as a patients co-morbidities. Although the use of protocols may aid decision making the final decision whether to perform an intervention lies with the physician and thus will be open to individual interpretation.

### Timing of intervention

The longer patients went without intervention the worse the outcome was, in the studies that analysed this however no statistically significant finding about the exact time was suggested in any of the papers. A large literature review of resuscitative thoracotomy found that outcomes were improved if the patient arrested in front of the physician performing the thoracotomy or in the recent past (eg en route to hospital) [46]. This finding is mirrored in the use of thoracotomy for thoracic trauma with universally poor outcomes the longer the patient is in cardiac arrest [4]. This correlates with findings that suggest that hypovolemic induced asystole is almost a universally un survivable event and thus the earlier cross clamping of the aorta and therefore control of haemorrhage can occur the higher the potential for survival [1]. However particularly in the peri-arrest patient is in important to note that intervention too early could have a detrimental effect on an already physiological deplete patient and a balance should be struck if possible. There is no guide as to an intervention cut off threshold such as a systolic blood pressure and decision for intervention must be made after a rapid thorough evaluation of the patient and based on the full clinical picture.

### Neurological outcome

Only 3 studies investigated or commented on the neurological outcome of survivors to discharge [31, 36, 37, 46]. A literature review of a much larger group of patients, but also more variety in indications and injuries, reported that 92.4% of survivors had no neurological deficit but did not quantify what exactly this meant [46]. Although in the UK in the year 2017 91% of patients with an injury severity score of over 9 survived to discharge their neurological status at that point was not recorded and thus makes comparison difficult [48]. Internationally one Australian study assessed 3824 patients who had an injury severity score of 15 or above 12 months after their injuries, 80% of patients reported some degree of neurological dysfunction [49]. The paediatric population suffering from a traumatic cardiac arrest has been assessed by a large systematic review which demonstrated as high as 44.3% of survivors had a good neurological outcome at discharge [50]. A large systematic review assessing neurological outcomes in 1369 patient undergoing thoracotomy for blunt traumatic cardiac arrest found 1.5% of patients survived with a good neurological outcome with vital signs being present either in the ED or on scene and CPR on going for no longer than 15 minutes [42].

### Weakness of study

The main weakness in this study was the lack of a control group for comparison with the intervention group. The outcomes of a patient with abdominal trauma and unrelenting shock either in, or close to, cardiac arrest not undergoing a resuscitative thoracotomy is largely unknown and thus expert opinion was used to provide comparison. The study therefore relies on this information being accurate to try and draw comparisons and thus conclusions, which, given the known bias associated with expert opinion questions the overall validity of the study. All the papers included were retrospective cohort studies with no randomisation and thus the potential for selection bias. This was potentially further compounded as the investigators were not blinded and therefore gives a risk of measurement bias. Although this is unlikely to be a problem when assessing 30 day mortality with the secondary outcomes of the study this may have had some bearing. The majority of the data collection occurred at large level 1 trauma centre’s within the united states. It is not unreasonable to question not only performance bias when compared to studies published outside the large trauma centre’s but also the applicability to the UK patient population and practices. Ideally a more controlled study should be performed with a clear indication for intervention with randomisation to groups with blinding of researchers to increase overall validity of the findings. This however would be a difficult if not impossible task. Practically, in the UK, a pre-alert from the ambulance service may be forth coming with accurate information about the patients en route to hospital however this is often not the case with key details that affect decision making being omitted [51]. In the pre-hospital environment information from the public is even more unreliable meaning randomisation decisions would need to be made at the time of patient assessment in a very stressful environment [52]. Although possible, timing would be important, and management would rely on the correct equipment and skills set being available immediately which would likely lead to low numbers of patients being recruited. There would also be ethical considerations when dealing with patients as randomised to the non-treatment group, based on the estimated survival probabilities, would also certainly be a death sentence. Alternatives to improve validity for future trials would be the use of case matching when conducting retrospective case reviews however in institutions with a clear thoracotomy policy, finding those that did not undergo the procedure would be challenging.

This study has a number of limitations when looking specifically at the research question. Although the primary aim of the study was to look at outcomes following abdominal trauma, patients suffering with polytrauma were also included. This decreases the overall validity of the findings particularly guiding application within the clinical environment when faced with a patient with isolated abdominal trauma. The exclusion of foreign language papers also has the potential to lead to missing data which when dealing with the small numbers described could have an influence on the conclusions drawn.

### Future developments

The use of resuscitative thoracotomy has been documented for many years however new technologies are in development. One of the aims of a thoracotomy in non-thoracic trauma is to cross clamp the descending aorta and achieve haemorrhage control. Resuscitative endovascular balloon occlusion of the aorta (REBOA) aims to achieve control of haemorrhage with a less invasive approach. Although it is in the early stage of development is has already been used in the pre-hospital environment as well as trauma centres within the UK [53]. However, studies are yet to show a demonstrable benefit when compared to resuscitative thoracotomy [54, 55]. One comparison study suggested a mortality benefit when compared to resuscitative thoracotomy however those undergoing REBOA were less haemodynamically unstable and REBOA took longer to perform [36].

Resuscitative thoracotomy for abdominal trauma is and will remain a controversial and divisive procedure. Although the evidence is of low quality it is difficult to see how higher levels of evidence, such as randomised control trials, will ever be put into place given the ethical implications and controversy surrounding practice. With experts suggesting a survival rate, with current management strategies, at best 1.7%. In the population in which trauma has the highest prevalence, namely young fit individuals, this is unacceptable when compared to medical cardiac arrest survival of 8.6%. Newer, more targeted interventions are in development however the current evidence is sparse and suggests at best equal outcomes when compared to thoracotomy.

## Conclusion

Although a highly controversial, invasive and arguably last-ditch effort procedure, pre-theatre thoracotomy should be considered in the peri-arrest or arrested patient with abdominal trauma either in isolation or as part of polytrauma. The best outcomes are achieved with patients not in cardiac arrest or who have recently arrested and who do not have a head injury present. The earlier the intervention can be performed, the better the outcome for patients, with survival figures of up to 18% with intervention compared to 0.001% without, however more high quality evidence is require to demonstrate a definite mortality benefit for patients.

## Supplementary information

**Additional file 1:** Appendix’s: Outcomes following resuscitative thoracotomy for abdominal exsanguination, a systematic review

## Data Availability

Separate uploaded document.
