# Supplementary material for: Outcomes following resuscitative thoracotomy for abdominal exsanguination, a systematic review
Source: Scand J Trauma Resusc Emerg Med. 2020 Feb 6;28:9. doi: 10.1186/s13049-020-0705-4 (PMC7006065; doi:10.1186/s13049-020-0705-4)
Supplement: Supplementary file 1 — Additional file 1: Appendix’s: Outcomes following resuscitative thoracotomy for abdominal exsanguination, a systematic review [file 13049_2020_705_MOESM1_ESM.zip › PE_corr/appendix SJ .docx]

**Appendix’s: Outcomes following resuscitative thoracotomy for abdominal exsanguination, a systematic review**

**Appendix 1**

Copy of search strategy used for Embase and Medline databases


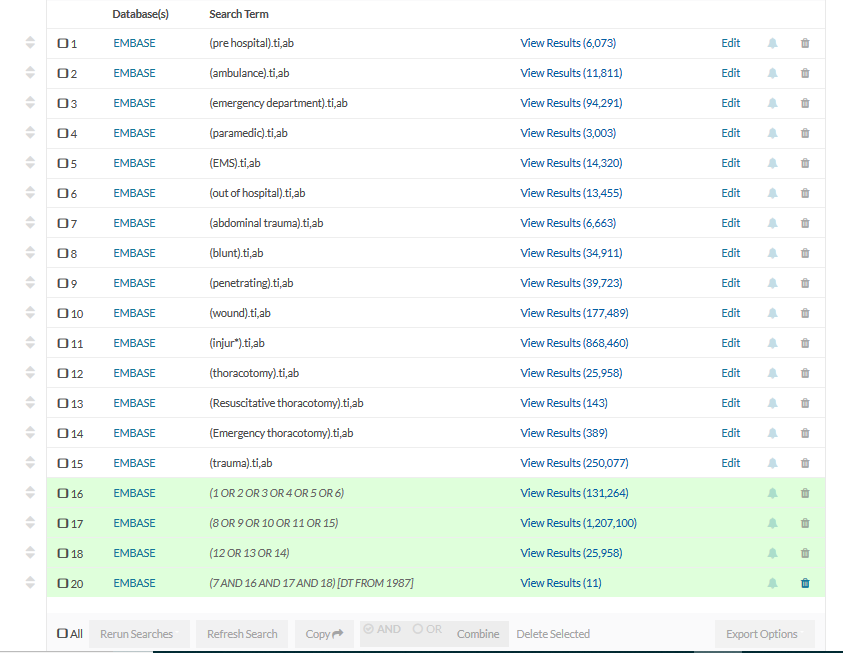


**Appendix 2**

Summary of inclusion and exclusion criteria a long with perceived weakness to study.

|  | Inclusion | Exclusion | Weakness |
| --- | --- | --- | --- |
| Pre-theatre | Procedure must be performed in the pre- theatre environment (ED or pre-hospital) | In theatre | Will generate significant amount of anecdotal evidence |
| Abdominal trauma | Must have stated injuries of patients based on either clinical assessment or in hospital / pre-hospital imaging | Isolated chest/ pelvis | Will include “poly trauma patients” thus reducing validity of review for isolated intra-abdominal injury |
| Outcomes | Must state outcomes of patients in terms of survival to destination or discharge. | No patient outcomes included |  |
| Thoracotomy | Any thoracotomy (clamshell / left lateral) | Non-thoracotomy interventions e.g. REBOA | Excludes novel and emerging techniques e.g. REBOA |

**Appendix 3**

| **Title** | **Inclusion, exclusion criteria and listed indication for thoracotomy** | **Study design** |
| --- | --- | --- |
| Velmahos 1995^[[1]](#endnote-1)^ | Inclusion criteria: All patients undergoing resuscitative thoracotomy in single department over specified time period  Exclusion criteria : Patients with no vital signs on arrival to ED  Patients deteriorating in ED (taken straight to theatre)  Indication for thoracotomy : Patient’s with agonal breathing  Patient with present pupillary reflex  Loss of signs of life around time of admission | Retrospective analysis of case records |
| Asensio 2003 ^[[2]](#endnote-2)^ | Inclusion criteria: Patients with iliac vessel injuries  Exclusion criteria: Not stated  Indications for thoracotomy: Not stated | Retrospective analysis of case records |
| Asensio 2005^[[3]](#endnote-3)^ | Inclusion: All patient presenting to institution with proven celiac axis injuries over study period.  Exclusion criteria: Not stated  Indications for thoracotomy: not stated | Retrospective analysis of case records |
| Blocksom 2004^[[4]](#endnote-4)^ | Inclusion criteria: All patients undergoing in single centre who had diagnosis of duodenal injury  Exclusion criteria: Not stated  Indications for thoracotomy: not stated | Retrospective analysis of case records |
| Kalina 2009^[[5]](#endnote-5)^ | Inclusion criteria : All patients undergoing thoracotomy in single centre  Exclusion criteria: Not stated  Indication for thoracotomy: Systolic below 70 mmHG or clinical decision | Retrospective analysis of case records |
| Moore 2016^[[6]](#endnote-6)^ | Inclusion: All patients undergoing thoracotomy during time period for trauma  Exclusion criteria: Not stated  Indications for thoracotomy: not stated | Retrospective analysis of case records |
| Asensio 2007^[[7]](#endnote-7)^ | Inclusion: All patient admitted to institution within time period with proven SMV injury  Exclusion criteria: Not stated  Indications for thoracotomy: Not stated | Retrospective analysis of case records |
| Ross 1988^[[8]](#endnote-8)^ | Inclusion: Any patient presenting to institution within time period and with documented systolic BP below 90 and suspected abdominal or pelvic injury.  Exclusion criteria: Not stated  Indications for thoracotomy: Not stated | Retrospective analysis of case reports |
| Nicholas 2003^[[9]](#endnote-9)^ | Inclusion: Patient undergoing emergency laparotomy during time period for penetrating trauma  Exclusion criteria: Negative findings at laparotomy  Multiple injuries  Indications for thoracotomy: Not stated | Retrospective analysis of case reports |
| Mazzorana 1994^[[10]](#endnote-10)^ | Inclusion: All patient’s undergoing thoracotomy within time period.  Exclusion criteria: Not stated  Indications for thoracotomy: Not stated | Retrospective analysis of case reports |
| Tyburski 2001^[[11]](#endnote-11)^ | Inclusion: All patients presenting to institution within time period.  Exclusion criteria: Not stated  Indications for thoracotomy: Not stated | Retrospective analysis of case reports |
| Moore 2015^[[12]](#endnote-12)^ | Inclusion: All patients undergoing REBOA or resuscitative thoracotomy over an 18 month period in 2 level one trauma centres  Exclusion criteria: Suspected or confirmed chest trauma  Indications for thoracotomy: Not stated | Retrospective analysis of trauma registry |
| Seamon 2008^[[13]](#endnote-13)^ | Inclusion: All patients undergoing emergency thoracotomy for abdominal trauma over 6 year period at single centre.  Exclusion criteria: Not stated  Indications for thoracotomy: dependant on clinican | Retrospective case review |
| Asensio 2001^[[14]](#endnote-14)^. | Inclusion: All patients presenting to level 1 trauma centre with one feature of 1) Estimated blood loss of over 2L intraoperatively  2) Required more than 1.5L of PRBC during resuscitation 3) diagnosis of exsanguination  Exclusion criteria: Not stated  Indications for thoracotomy: clinical discretion | Retrospective case series |
| Asensio 2000^[[15]](#endnote-15)^. | Inclusion: Patients with abdominal vascular injury  Exclusion criteria: Not stated  Indication for thoracotomy : unrelenting shock, cardiac arrest or severe haemodynamic compromise | Retrospective case series |
| Branney 1998^[[16]](#endnote-16)^. | Inclusion:  Any patient presenting to ED without a palpable pulse or absent blood pressure. Patient’s not responding to resuscitation with a systolic BP below 70.  Exclusion criteria: Not stated  Indications for thoracotomy: Not stated | Retrospective case series |
| Lustenberger 2012.^[[17]](#endnote-17)^ | Inclusion: All patients presenting to single trauma centre during time period  Exclusion criteria: no resuscitative thoracotomy  Indication for thoracotomy: non-recordable  blood pressure in patients with penetrating injury who  had signs of life en route or at the scene; patients with blunt  trauma who lost signs of life in the hospital or immediately  before arrival; and exsanguinated patients with no immediate response to fluid resuscitation | Retrospective case review in one centre |

**Appendix 4**

A table summarises the Study, description of participants study design, outcome measure, results and conclusion for each analysed study

| **Study** | **Description of participants** | **Study design** | **Outcomes** | **Results** | **Conclusion** |
| --- | --- | --- | --- | --- | --- |
| Velmahos 1995^[[18]](#endnote-18)^ | 118 abdomen, 501 poly trauma, ED attenders.  Excluded all those without vital signs on arrival to ED (except agonal breathing and pupil reflex)  Loss of vital signs “around time of admission”. Those deteriorating taken to theatre | Retrospective analysis of case records | Survival, complications, neurological defect | 8 isolated abdominal injury survivors (6.8%)  One polytrauma survivor | Survival for penetrating abdominal trauma 6.8%. Best outcomes with witnessed loss of signs of life (6.8%) |
| Asensio 2003 ^[[19]](#endnote-19)^ | 100-month period of all patients attending single centre with iliac vessel injuries (combination of penetrating and blunt) | Retrospective analysis of case records | Survival and mortality | 22 patients undergoing ED thoracotomy with 18% survival rate | Pre-theatre thoracotomy for iliac vessel injury survival of 18% |
| Asensio 2005^[[20]](#endnote-20)^ | 132 months all patient with proven celiac axis injury | Retrospective analysis of case records | Mortality | 3 patients undergoing ED thoracotomy – survived to theatre but not to discharge | Pre-theatre thoracotomy no survival |
| Blocksom 2004^[[21]](#endnote-21)^ | 22 years all patients undergoing emergency laparotomy in single centre | Retrospective analysis of case records | Survivors more than 48 hours | 27 patients undergoing ED thoracotomy (24 died)  195 no ED thoracotomy 26 died | 11.1 % survival |
| Kalina 2009^[[22]](#endnote-22)^ | All patient’s undergoing resuscitative thoracotomy for blunt trauma and penetrating trauma in a single centre. Included thoracic trauma patients | Retrospective analysis of case records | Survival | Seven abdominal and 13 patients with multiple injuries.  Presence of SOL in field best predictor of survival however grouped thoracic injuries with abdominal injuries | Unclear as to who were survivors based on location of injury |
| Moore 2016^[[23]](#endnote-23)^ | All patient’s attending a single centre who underwent emergency department thoracotomy   - Isolated abdomen - 116 - Multiple (no chest, no head injury) 91 - Multiple (no chest, head) 99 - Multiple (chest, no head) 355 - Multiple (chest and head) 458 | Retrospective analysis of case records | Survival, neurological outcome, impact of pre-hospital CPR | Isolated abdomen -6% survival (stab with CPR), 9% blunt no CPR  No survival in isolated abdomen if no CPR in gunshot and stab  No survival with CPR in isolated abdomen if blunt or gunshot.  10%. Survival if Blunt and no CPR  8% survival if stab with CPR  68% no permanent neurological deficit with 12% mild neurological deficit and remaining 20% in a persistent vegetative state | Better outcomes if isolated penetrating abdominal injury and no pre-hospital CPR (5%)  Blunt isolated abdomen and no CPR 2% |
| Asensio 2007^[[24]](#endnote-24)^ | 51 patients with proven superior mesenteric vein injuries over a 156 month period to a single centre | Retrospective analysis of case records | Survival | 4 patients underwent pre-theatre thoracotomy – non survived | 0% survival in pre-theatre thoracotomy |
| Ross 1988^[[25]](#endnote-25)^ | 37 patients presenting with severe hypovolemic shock secondary to sub diaphragmatic injuries to a single centre over a 12-month period | Retrospective analysis of case reports | Survival | 7 patients underwent pre-theatre thoracotomy (for hypovolemic cardiac arrest) – non survived | 0% SURVIVAL IN hypovolemic cardiac arrest |
| Nicholas 2003^[[26]](#endnote-26)^ | 250 patients undergoing emergency laparotomy for penetrating abdominal injury over a three-year period in a single centre. 7 pre-theatre thoracotomies | Retrospective analysis of case reports | Survival | 7 patients underwent pre-theatre thoracotomy | 0% survival in 7 patients. |
| Mazzorana 1994^[[27]](#endnote-27)^ | 252 patients undergoing pre-theatre thoracotomy in a single centre over a 5 year period. | Retrospective analysis of case reports | Survival,  Signs of life pre-hospital and ED, neurological status | Four patients with combined abdominal /thoracic penetrating trauma survived neurologically intact.  No blunt trauma patients survived  No patients survived with neurological deficit.  Signs of life present in ED or lost just before ED were a predictor of survival | Four patients survived pre-theatre thoracotomy neurological intact. Signs of life at time of thoracotomy were best indicator of survival |
| Tyburski 2001^[[28]](#endnote-28)^ | 470 patients attending a single centre with confirmed abdominal vascular injury  31 patients underwent pre-theatre thoracotomy for systolic BP below 70 and arrest or rapidly deteriorating clinical condition | Retrospective analysis of case reports | Mortality | 31 patients underwent pre-theatre thoracotomy with 6% survival  Pre-laparotomy thoracotomy (but performed in theatre) 51 patients with 29 “responders” (increase in BP to above 90 systolic) to aortic cross clamping surviving. Compared to those who did not have pre-laparotomy thoracotomy and cross clamping in theatre (10.5% survival) there was a statically significant difference with advantage to pre-laparotomy thoracotomy (p 0.01)  Initial ED Blood pressure independent major prognostic factor. Systolic of 70 mm HG the survival rate was 76% | 6% survival with pre-theatre thoracotomy  38% survival with pre-laparotomy thoracotomy and response to aortic cross clamping.  Better outcomes with systolic over 70 mm HG on arrival to ED. |
| Moore 2015^[[29]](#endnote-29)^ | 72 patients undergoing resuscitative thoracotomy and 24 patients undergoing REBOA for exsanguinating haemorrhage originating from the abdomen or pelvis | Retrospective analysis of trauma registry | In hospital mortality | 90.3% resuscitative thoracotomy patients died with 69.2% in ED, 9.2% in theatre and 21.6% in ICU  14 patients who survived to ICU 71.4% died of haemorrhage, 2 died of multiple organ failure, 2 died from head injury.  Majority of RT patients discharged to rehabilitation hospital (57.1%) or skilled nursing facility (14.4%) | Unable to separate abdominal and pelvic injuries.  9.7% survival with pre-theatre thoracotomy to discharge with 28.5% chance of no neurological deficit. |
| Seamon 2008^[[30]](#endnote-30)^ | 50 patients undergoing pre-laparotomy emergency department thoracotomy for abdominal exsanguination attending one centre over a 6 year period | Retrospective case review | Survival to discharge | Overall survival of 16% to discharge with normal neurological function. | 16% survival to discharge with normal neurological function |
| Asensio 2001^[[31]](#endnote-31)^. | 180 patients undergoing EDT for exsanguination.  Patients surviving EDT then combined with those having intraoperative resuscitative thoracotomy | Retrospective case series | Survival | 180 patients underwent EDT.  99 patients (55%) did not survive to theatre.  All those undergoing either EDT or ODT 28% survived to discharge  Independent survival factor – spontaneous breathing in ED | 45% survival to theatre with Pre-theatre thoracotomy  Grouped with in theatre pre-laparotomy thoracotomy survival 28% to discharge |
| Asensio 2000^[[32]](#endnote-32)^. | 43 patients undergoing emergency department thoracotomy for abdominal vascular injury and presence of unrelenting shock, cardiac arrest or severe haemodynamic compromise | Retrospective case series | Survival | 1 survivor to discharge (2%)  Overall mortality 54% (of all 302 patients in series) | 2% survival in patients requiring EDT for abdominal vascular injuries |
| Branney 1998^[[33]](#endnote-33)^. | 868 EDT’s performed over a 23-year period  Indications for EDT are patient without palpable pulse or blood pressure. Some patients with a systolic below 70 not responding to resuscitation. | Retrospective case series | Survival, neurological outcome | Stab abdominal 17, 1 survivor (5%)  Gunshot abdomen 56, 7 survivors (13%)  Blunt abdomen 51, 1 survivor 2%.  All above neurologically intact  9.5% survival rate for all patients if vital signs present in field vs 1.9% if absent.  Time from paramedic dispatch to EDT was not statistically significant (25 Vs 27.2 minutes) | 5% survival in abdominal stab wounds, 13% in gunshot abdomen, 2% in blunt trauma in patient who are in cardiac arrest. |
| Lustenberger 2012.^[[34]](#endnote-34)^ | 31 patients with non-recordable  blood pressure in patients with penetrating injury who  had signs of life en route or at the scene; patients with blunt  trauma who lost signs of life in the hospital or immediately  before arrival; and exsanguinated patients with no immediate response to fluid resuscitation | Retrospective case review in one centre | 31 patients with abdominal injuries undergoing EDT.  11 severe liver injury  13 splenic rupture  4 kidney rupture  3 intrabdominal aortic laceration | 31 patients with abdominal injuries  11 liver injuries 2 survivors (18%)  13 splenic injuries 1 survivor (8%)  4 renal injuries no survivors  3 aortic injury 1 survivor (33%) | 13% survival in 31 patients. |

**References**

1. Velmahos GC, Degiannis MD, Allwood AC, Saadia R. Outcome of a strict policy on emergency department thoracotomies. Arch Surg. 1995;130:774-7. [↑](#endnote-ref-1)
2. Asensio JA, Petrone P, Roldán G, Kuncir E, Rowe VL, Chan L, Shoemaker W, Berne TV. Analysis of 185 iliac vessel injuries: risk factors and predictors of outcome. Archives of Surgery. 2003 Nov 1;138(11):1187-94. [↑](#endnote-ref-2)
3. Asensio JA, Petrone P, Kimbrell B, Kuncir E. Lessons learned in the management of thirteen celiac axis injuries. Southern medical journal. 2005 Apr 1;98(4):462-7. [↑](#endnote-ref-3)
4. Blocksom JM, Tyburski JG, Sohn RL, Williams M. Prognostic Determinants in Duodenal Injuries/DISCUSSION. The American Surgeon. 2004 Mar 1;70(3):248. [↑](#endnote-ref-4)
5. Kalina M, Teeple E, Fulda G. Are there still selected applications for resuscitative thoracotomy in the emergency department after blunt trauma?. Delaware medical journal. 2009 May;81(5):195-8. [↑](#endnote-ref-5)
6. Moore HB, Moore EE, Burlew CC, Biffl WL, Pieracci FM, Barnett CC, Bensard DD, Jurkovich GJ, Fox CJ, Sauaia A. Establishing benchmarks for resuscitation of traumatic circulatory arrest: success-to-rescue and survival among 1,708 patients. Journal of the American College of Surgeons. 2016 Jul 1;223(1):42-50. [↑](#endnote-ref-6)
7. Asensio JA, Petrone P, Garcia-Nuñez L, Healy M, Martin M, Kuncir E. Superior mesenteric venous injuries: to ligate or to repair remains the question. Journal of Trauma and Acute Care Surgery. 2007 Mar 1;62(3):668-75. [↑](#endnote-ref-7)
8. Ross SE, Schwab CW. Resuscitation of subdiaphragmatic exsanguination. The American surgeon. 1988 Apr;54(4):200-3. [↑](#endnote-ref-8)
9. Nicholas JM, Rix EP, Easley KA, Feliciano DV, Cava RA, Ingram WL, Parry NG, Rozycki GS, Salomone JP, Tremblay LN. Changing patterns in the management of penetrating abdominal trauma: the more things

   change, the more they stay the same. Journal of Trauma and Acute Care Surgery. 2003 Dec 1;55(6):1095-110 [↑](#endnote-ref-9)
10. Mazzorana V, Smith RS, Morabito DJ, Brar HS. Limited utility of emergency department thoracotomy. The American surgeon. 1994 Jul;60(7):516-20. [↑](#endnote-ref-10)
11. Tyburski JG, Wilson RF, Dente C, Steffes C, Carlin AM. Factors affecting mortality rates in patients with abdominal vascular injuries. Journal of Trauma and Acute Care Surgery. 2001 Jun 1;50(6):1020-6. [↑](#endnote-ref-11)
12. Moore LJ, Brenner M, Kozar RA, Pasley J, Wade CE, Baraniuk MS, Scalea T, Holcomb JB. Implementation of resuscitative endovascular balloon occlusion of the aorta as an alternative to resuscitative thoracotomy for noncompressible truncal hemorrhage. Journal of Trauma and Acute Care Surgery. 2015 Oct 1;79(4):523-32 [↑](#endnote-ref-12)
13. Seamon MJ, Pathak AS, Bradley KM, Fisher CA, Gaughan JA, Kulp H, Pieri PG, Santora TA, Goldberg AJ. Emergency department thoracotomy: still useful after abdominal exsanguination?. Journal of Trauma and Acute Care Surgery. 2008 Jan 1;64(1):1-8. [↑](#endnote-ref-13)
14. Asensio JA, McDuffie L, Petrone P, Roldán G, Forno W, Gambaro E, Salim A, Demetriades D, Murray J, Velmahos G, Shoemaker W. Reliable variables in the exsanguinated patient which indicate damage control and predict outcome. The American journal of surgery. 2001 Dec 1;182(6):743-51. [↑](#endnote-ref-14)
15. Asensio JA, Chahwan S, Hanpeter D, Demetriades D, Forno W, Gambaro E, Murray J, Velmahos G, Marengo J, Shoemaker WC, Berne TV. Operative management and outcome of 302 abdominal vascular injuries. The American journal of surgery. 2000 Dec 1;180(6):528-34. [↑](#endnote-ref-15)
16. Branney SW, Moore EE, Feldhaus KM, Wolfe RE. Critical analysis of two decades of experience with postinjury emergency department thoracotomy in a regional trauma centre. Journal of Trauma and Acute Care Surgery. 1998 Jul 1;45(1):87-94. [↑](#endnote-ref-16)
17. Lustenberger T, Labler L, Stover JF, Keel MJ. Resuscitative emergency thoracotomy in a Swiss trauma centre. British journal of surgery. 2012 Apr 1;99(4):541-8. [↑](#endnote-ref-17)
18. Velmahos GC, Degiannis MD, Allwood AC, Saadia R. Outcome of a strict policy on emergency department thoracotomies. Arch Surg. 1995;130:774-7. [↑](#endnote-ref-18)
19. Asensio JA, Petrone P, Roldán G, Kuncir E, Rowe VL, Chan L, Shoemaker W, Berne TV. Analysis of 185 iliac vessel injuries: risk factors and predictors of outcome. Archives of Surgery. 2003 Nov 1;138(11):1187-94. [↑](#endnote-ref-19)
20. Asensio JA, Petrone P, Kimbrell B, Kuncir E. Lessons learned in the management of thirteen celiac axis injuries. Southern medical journal. 2005 Apr 1;98(4):462-7. [↑](#endnote-ref-20)
21. Blocksom JM, Tyburski JG, Sohn RL, Williams M. Prognostic Determinants in Duodenal Injuries/DISCUSSION. The American Surgeon. 2004 Mar 1;70(3):248. [↑](#endnote-ref-21)
22. Kalina M, Teeple E, Fulda G. Are there still selected applications for resuscitative thoracotomy in the emergency department after blunt trauma?. Delaware medical journal. 2009 May;81(5):195-8. [↑](#endnote-ref-22)
23. Moore HB, Moore EE, Burlew CC, Biffl WL, Pieracci FM, Barnett CC, Bensard DD, Jurkovich GJ, Fox CJ, Sauaia A. Establishing benchmarks for resuscitation of traumatic circulatory arrest: success-to-rescue and survival among 1,708 patients. Journal of the American College of Surgeons. 2016 Jul 1;223(1):42-50. [↑](#endnote-ref-23)
24. Asensio JA, Petrone P, Garcia-Nuñez L, Healy M, Martin M, Kuncir E. Superior mesenteric venous injuries: to ligate or to repair remains the question. Journal of Trauma and Acute Care Surgery. 2007 Mar 1;62(3):668-75. [↑](#endnote-ref-24)
25. Ross SE, Schwab CW. Resuscitation of subdiaphragmatic exsanguination. The American surgeon. 1988 Apr;54(4):200-3. [↑](#endnote-ref-25)
26. Nicholas JM, Rix EP, Easley KA, Feliciano DV, Cava RA, Ingram WL, Parry NG, Rozycki GS, Salomone JP, Tremblay LN. Changing patterns in the management of penetrating abdominal trauma: the more things

    change, the more they stay the same. Journal of Trauma and Acute Care Surgery. 2003 Dec 1;55(6):1095-110 [↑](#endnote-ref-26)
27. Mazzorana V, Smith RS, Morabito DJ, Brar HS. Limited utility of emergency department thoracotomy. The American surgeon. 1994 Jul;60(7):516-20. [↑](#endnote-ref-27)
28. Tyburski JG, Wilson RF, Dente C, Steffes C, Carlin AM. Factors affecting mortality rates in patients with abdominal vascular injuries. Journal of Trauma and Acute Care Surgery. 2001 Jun 1;50(6):1020-6. [↑](#endnote-ref-28)
29. Moore LJ, Brenner M, Kozar RA, Pasley J, Wade CE, Baraniuk MS, Scalea T, Holcomb JB. Implementation of resuscitative endovascular balloon occlusion of the aorta as an alternative to resuscitative thoracotomy for noncompressible truncal hemorrhage. Journal of Trauma and Acute Care Surgery. 2015 Oct 1;79(4):523-32 [↑](#endnote-ref-29)
30. Seamon MJ, Pathak AS, Bradley KM, Fisher CA, Gaughan JA, Kulp H, Pieri PG, Santora TA, Goldberg AJ. Emergency department thoracotomy: still useful after abdominal exsanguination?. Journal of Trauma and Acute Care Surgery. 2008 Jan 1;64(1):1-8. [↑](#endnote-ref-30)
31. Asensio JA, McDuffie L, Petrone P, Roldán G, Forno W, Gambaro E, Salim A, Demetriades D, Murray J, Velmahos G, Shoemaker W. Reliable variables in the exsanguinated patient which indicate damage control and predict outcome. The American journal of surgery. 2001 Dec 1;182(6):743-51. [↑](#endnote-ref-31)
32. Asensio JA, Chahwan S, Hanpeter D, Demetriades D, Forno W, Gambaro E, Murray J, Velmahos G, Marengo J, Shoemaker WC, Berne TV. Operative management and outcome of 302 abdominal vascular injuries. The American journal of surgery. 2000 Dec 1;180(6):528-34. [↑](#endnote-ref-32)
33. Branney SW, Moore EE, Feldhaus KM, Wolfe RE. Critical analysis of two decades of experience with postinjury emergency department thoracotomy in a regional trauma center. Journal of Trauma and Acute Care Surgery. 1998 Jul 1;45(1):87-94. [↑](#endnote-ref-33)
34. Lustenberger T, Labler L, Stover JF, Keel MJ. Resuscitative emergency thoracotomy in a Swiss trauma centre. British journal of surgery. 2012 Apr 1;99(4):541-8. [↑](#endnote-ref-34)
