# Supplementary material for: Outcomes following resuscitative thoracotomy for abdominal exsanguination, a systematic review
Source: Scand J Trauma Resusc Emerg Med. 2020 Feb 6;28:9. doi: 10.1186/s13049-020-0705-4 (PMC7006065; doi:10.1186/s13049-020-0705-4)
Supplement: Supplementary file 1 — Additional file 1: Appendix’s: Outcomes following resuscitative thoracotomy for abdominal exsanguination, a systematic review [file 13049_2020_705_MOESM1_ESM.zip › PE_corr/Dear Ryan.pdf]

Dear Ryan,

Thank you for your emails and apologies for the delay.
I attach the missing file with all the appendices here.
Please do let me know if you need further assistance with this article.

Kind regards,

Lydia Georgiadou
Production Editor

BMC

4 Crinan Street
London N1 9XW
